# Drought and nutrient pollution produce multiple interactive effects in stream ecosystems

**DOI:** 10.1371/journal.pone.0269222

**Published:** 2022-07-14

**Authors:** Robert J. Fournier, Daniel D. Magoulick

**Affiliations:** 1 Department of Environmental Science, Policy, and Management, University of California, Berkeley, California, United States of America; 2 Department of Biological Sciences, U.S. Geological Survey, Arkansas Cooperative Fish and Wildlife Research Unit, University of Arkansas, Fayetteville, Arkansas, United States of America; University of Eldoret, KENYA

## Abstract

Drought and nutrient pollution can affect the dynamics of stream ecosystems in diverse ways. While the individual effects of both stressors are broadly examined in the literature, we still know relatively little about if and how these stressors interact. Here, we performed a mesocosm experiment that explores the compounded effects of seasonal drought via water withdrawals and nutrient pollution (1.0 mg/L of N and 0.1 mg/L of P) on a subset of Ozark stream community fauna and ecosystem processes. We observed biological responses to individual stressors as well as both synergistic and antagonistic stressor interactions. We found that drying negatively affected periphyton assemblages, macroinvertebrate colonization, and leaf litter decomposition in shallow habitats. However, in deep habitats, drought-based increases in fish density caused trophic cascades that released algal communities from grazing pressures; while nutrient enrichment caused bottom-up cascades that influenced periphyton variables and crayfish growth rates. Finally, the combined effects of drought and nutrient enrichment interacted antagonistically to increase survival in longear sunfish; and stressors acted synergistically on grazers causing a trophic cascade that increased periphyton variables. Because stressors can directly and indirectly impact biota—and that the same stressor pairing can act differentially on various portions of the community simultaneously—our broad understanding of individual stressors might not adequately inform our knowledge of multi-stressor systems.

## Introduction

Biodiversity is declining at an unprecedented rate [1] with broad impacts to ecosystem functioning [2]. This decline is pronounced in aquatic systems, and freshwater biota are among the most threatened globally [3,4]. In stream systems, anthropogenic actions have exacerbated biodiversity loss, and systems in North America are especially threatened [5,6]. Two major stressors of these systems, drought and nutrient pollution, can impose a diverse array of ecological effects on stream communities. While both stressors are broadly examined in the existing literature [7,8], studies that examine their combined effects remain relatively rare (but see [9,10]). Stressor interactions are often complex as they can act additive, synergistically, or antagonistically [11] and initiate regime shifts if ecological degradation is sufficiently high [12,13].

Freshwater systems are particularly vulnerable to the effects of multiple stressors because their inherent heterogeneity might interact with a variety of disturbance events [14,15]. Most examinations of multiple stressors are experimental in nature and focus largely on population-level responses [16]. Approximately 41% of documented stressor interactions in freshwater systems are antagonistic and may not affect diversity or functioning metrics, while stressor interactions that produce synergistic or additive effects account for 28% and 16% of studies, respectively [16]. Additionally, population variables are more likely to display additive or synergistic responses to multiple stressors than metrics that explain community or ecosystem-level processes [9,10,17]. Because freshwater systems are susceptible to ecological degradation brought on by multiple stressors, and this vulnerability is expected to increase as human population and resource use grows [18,19], more work must be done to disentangle the effects of multiple stressors in stream ecosystems [20].

Drought affects aquatic ecosystems on every continent and can greatly influence stream population and community dynamics [21–23]. In addition to direct mortality brought on by receding waters, aquatic ecosystems undergo several physical, chemical, and hydrological changes during drying that can elicit direct biological responses [24]. In systems like the Ozark Highlands of Arkansas, Missouri, and Oklahoma, streams tend to dry in the late summer and early fall [25]. Unlike press-style supraseasonal droughts, seasonal droughts in these systems tend to pulse their effects in a relatively short period of time before returning to normal flow conditions [26,27]. During seasonal droughts, aquatic species must use refuge habitats in persistent waters or perish [7]. The riffle-pool geomorphology of these streams often allows pools to remain watered during seasonal drying events and serve as refuges [28,29]. However, the density of organisms increases when they are confined to refuges, amplifying the relative strength of biotic interactions and leaving biota susceptible to additional disturbances [24]. While drying constitutes a significant stressor to fish [7], invertebrate [30,31], and algal [32,33] populations, many species evolved adaptations to help them persist in drought-prone systems [7,12,25,34–36].

Nutrient enrichment represents one of the most severe threats to global freshwater biodiversity [37] and is often the single greatest cause of pollution in aquatic systems [38,39]. Anthropogenic input of inorganic nutrients via agriculture, urbanization, or the burning of fossil fuels can have bottom-up trophic effects on stream ecosystems as algal and microbial communities can be limited in their growth by available nitrogen and/or phosphorouss [40,41]. In lower order streams, benthic algae are often the primary drivers of autochthonous primary production [42]. When algal growth is unchecked by nutrient limitation, water quality can be diminished via decreased dissolved oxygen levels (resulting in fish kills) or algal-mediated toxins [43]. Under extreme levels of pollution, an overabundance of algal biomass can decouple trophic relationships and destabilize food web dynamics [44]. Nutrient enrichment can also directly stimulate bacterial and fungal growth—increasing detrital decomposition rates and secondary production [45–47]. Additionally, if systems are enriched over long time periods, communities might become increasingly homogenized across local (α diversity) and regional (β diversity) scales with corresponding ecological and evolutionary consequences [48,49].

While both seasonal drought and nutrient pollution can influence stream ecosystem dynamics individually, these stressors might interact in ecologically meaningful ways [19]. As water levels decrease throughout a system, habitats are more prone to extreme physical environmental conditions including spikes in temperature and dissolved oxygen levels [50]. When the stream is fully wetted, churning surface flow helps to mediate levels of dissolved oxygen and the potential effects of eutrophication via nutrient inputs can be counteracted [24]. However, the comparatively stagnant water present during droughts might become choked by algal growth, compounding eutrophic processes [24]. Furthermore, drought can potentially alter cycling patterns as nutrients become more concentrated [17,51]. As organismal density increases in drought conditions, the amplified strength of biotic interactions could have cascading consequences that facilitate or reduce algal growth [52] depending on food web structure. Additionally, the respiratory needs of organisms confined to refuges might exceed the dissolved oxygen levels present in eutrophic systems. Blooms of harmful algal species might also be of concern during droughts as toxins become increasingly concentrated as water volume decreases. In systems with significant agricultural use, irrigation-mediated water withdrawals can increase drought frequency and severity [53]. As agricultural introduction of nitrogen and phosphorous to ground and surface waters accounts for a significant amount of inland eutrophic systems [54], it is increasingly likely that drought and nutrient stressors co-occur. Finally, stream ecosystems with significant nutrient pollution are susceptible to additional stressors including fine sediment deposition and higher variations in water temperature [55], and nutrients might influence community resilience in systems that experience regular hydrological variation [17,56].

Despite a large body of established literature exploring the individual effects of drought, drying, [24,34], and nutrient pollution [8], the fine-scale effects of multiple stressors on stream communities remain underexplored [9,16]. Here, we performed a manipulative experiment that explored the effects of seasonal drought and nutrient pollution on stream ecosystems in an attempt to detangle potential stressor interactions. We hypothesize that drought will negatively affect fish and crayfish species via eutrophication—either by a decrease in growth or survivorship—and that drought will amplify the detrimental effects (e.g., decreased dissolved oxygen levels) of nutrient pollution on fish species. Additionally, we predict that nutrients will increase algal growth with potential cascading influences. However, we also expect that concentration effects in drought systems will help to stimulate algal production, potentially causing bottom-up trophic cascades and influencing overall system primary production. We also expect drought to decrease aspects of stream ecosystem functioning including leaf decomposition and invertebrate densities, however, these might be offset by positive effects of nutrient enrichment on photosynthetic portions of the food web. Finally, we expect that drought will affect the shallow and deep portions of the habitat differentially. Because aquatic ecosystems often experience anthropogenic degradation that includes multiple stressors, exploration of the ecology of these systems represents a necessary advancement in our understanding of disturbance ecology.

## Methods

### Experimental design

To test the effects of seasonal drought and nutrient pollution on stream ecosystem structure and function, we performed a fully factorial mesocosm experiment at the University of Arkansas biological greenhouse in the summer of 2017. Response variables included growth and survival of fish and crayfish species, chlorophyll a concentration, periphyton ash-free dry mass (AFDM), autotrophic index, sediment levels, and macroinvertebrate density. We also looked at measures of ecosystem functioning including net primary production and leaf litter decomposition.

Each mesocosm consisted of a 416 L oval polyethylene tank (1.26 m long × 0.84 m wide × 0.49 m deep) with a mixed substratum of gravel and pebbles [see 57]. Substratum was placed along a slope ranging from 0.10 to 0.32 m from the bottom of the tank so that ~⅓ of the benthic habitat was level and shallow (riffle), ~⅓ was sloped, and ~⅓ was level and deep (pool). Tanks were filled with municipal water and circulated by canister aquarium filters (Fluval 205 and 206; Hagen, Quebec, Canada). Filters provided flow-based agitation and likely provided minimal supplemental aeration. While additional aeration might affect some response variables, it was necessary to prevent complete fish and crayfish mortality early in the experiment. On May 26^th^, three weeks prior to the experiment, a 2 L slurry of scrubbed periphyton and stream water taken from Dye creek, Arkansas (35.94189,-94.18368) was added to each tank to facilitate algal and bacterial growth. Invertebrates were allowed to colonize the tanks naturally. Previous experiments have shown that Chironomidae readily colonize the mesocosms—both with and without lids—within the experimental time-frame (i.e., three weeks of colonization time; [52,57,58]. Fiberglass mesh (1 mm by 1 mm) lids were placed over each of the tanks and secured with clips to prevent escape.

Seasonal drought treatments consisted of water withdrawals over 3 days at a rate of 0.08 m/day until water was 0.25 m above the bottom of the deep end of the tanks. In these treatments, the substrate surface in the shallow portion of the tank was completely above the water line and remained so for the remainder of the experiment, while the deep portion remained wetted. Nutrient treatments involved enriching tanks to 1.0 mg/L of N (via the addition of NaNO_3_) and 0.1 mg/L of P (via the addition of Na_2_PO_4_) once at the beginning of the experiment [Sensu 59]. This initial enrichment was designed to create differential starting conditions rather than a maintained, constant level of nutrient concentrations. We housed the mesocosms in a climate-controlled bay under natural light in a 4 × 7 grid. Each of the four treatments—drought, nutrient enrichment, both stressors, and a no stressor control—had 7 replicates. Treatments were interspersed among the grid with a randomized treatment starting each row.

Focal species selected for the mesocosm experiments represent a cross-section of Ozark stream community fauna and occupy multiple ecological and trophic roles; longear sunfish (*Lepomis megalotis*), an insectivorous mesopredator, central stoneroller (*Campostoma anomalum*), a primarily algivorous minnow, and ringed crayfish (*Faxonius neglectus*), an omnivore. In Ozark streams, longear sunfish prefer pool habitats while central stonerollers primarily inhabit riffles and runs [60]. The ringed crayfish is a substantial driver of freshwater ecosystem structure and functioning as both producers and omnivorous consumers of biomass [61,62]. All species are distributed widely though the focal region and co-occur naturally in the Ozark Highlands [60,63]. Central stonerollers and longear sunfish were collected via backpack electrofishing from Scull and Mud creek in northwest Arkansas, respectively (36.06303, -94.09446; 36.120277, -94.153912). Ringed crayfish were collected via kick seining from Tanyard Creek, Arkansas (36.475565, -94.254442). Each mesocosm contained 18 individuals; 3 sunfish, 10 stonerollers, and 5 crayfish. These ratios coincide with naturally observed densities during flowing conditions [58]. Length (total length for fish (TL) and carapace length for crayfish (CL) and mass of each individual was recorded, and initial mean size for each species in all mesocosms was calculated. Initial mean size was similar for all treatments (~4.5 ± 2.6 g for stonerollers, ~17.5 ± 15g for longear sunfish, ~5.5 ± 3.5g for ringed crayfish). If at any point during the experiment an organism appeared to be in distress (e.g., floating on side, apparent injury), they were immediately euthanized by cervical dislocation or freezing. Organisms were monitored by personnel trained in IACUC animal handling guidelines several times daily. Organisms that died resulting from experimental conditions were removed from the tank and cataloged when found. Central stonerollers, longear sunfish, and ringed crayfish were added to the tanks on June 16^th^ (day 1) and allowed to acclimate for 5 days before water withdrawals for drought treatments began. Fish and crayfish were removed from the tanks on July 21^st^ (35 days total) and mean length and mass were recorded. This study was performed under the auspices of the University of Arkansas Animal Care and Use Committee protocol #16055.

Four 11 × 11 cm unglazed ceramic tiles were placed into each mesocosm for measurement of algal biomass and invertebrate colonization. No algae or invertebrates were on the tiles prior to the experiment. Two tiles were placed into the permanently watered section of each mesocosm, and two were placed in the shallow end. Leaves were collected from a local sugar maple (*Acer saccharum*), air dried to constant weight, and assembled into four, 3-g bags (32 cm by 22 cm with ~2.5-mm mesh, Volm Companies, Antigo, Wisconsin) and two each were placed in the shallow (riffle) and deep (pool) habitats. Two, 7-cm long slits were cut into each bag to allow for crayfish access. Tiles and leaf packs were removed on July 21^st^. We calculated net primary production by taking dissolved oxygen and temperature readings using a YSI multiparameter sonde (YSI Incorporated, Yellow Springs, Ohio) every 2 hours for 24 hours starting at 6am on July 17^th^ [Sensu 64]. While the aeration provided by our tanks might influence NPP readings when compared to natural systems, our experimental design aerated all tanks equally, and thus treatment effects on NPP might still be evident [52,65].

### Laboratory methods

Periphyton scrubbed from the tiles at the conclusion of the experiment was diluted with a known quantity of water and mixed until homogeneous before a 10 mL subsample was removed and filtered onto pre-weighed glass fiber filters (Pall GF/F) and frozen until analysis. As macroinvertebrate larvae largely remain intact after scrubbing, the remainder of the sample was searched for Chironomidae and density was calculated as individuals per cm^2^.The filter was placed in 10 mL of 95% ethanol for extraction and spectrophotometric analysis of chlorophyll a concentration. The contents of the cuvette were evacuated back onto the filter and then dried for 24 hours at 100°C to obtain dry mass before being combusted at 550°C for 3 hours, rewetted, and dried to obtain sediment organic matter as ash-free dry mass (AFDM). Leafpacks were air dried and weighed to determine change in mass. Autotrophic index was calculated as the ratio of chlorophyll a to AFDM. Sediment levels were calculated as the mass of the filtered slurry after ashing.

### Statistical analysis

Two-way analysis of variance (ANOVA) was used to assess the effects of drought and nutrient treatments on mean growth and survival of fish and crayfish, algal biomass, chironomid densities, sediment, and autotrophic index, net primary production, and leafpack decomposition. Here, we primarily focused on broad classifications of multiple stressor interactions (i.e., synergistic or antagonistic). As such, examination of the interaction term within ANOVAs is an appropriate method to examine directional multiple stressor interactions [66,67]. If there was no significant interaction between nutrient and drought treatments, one-way ANOVAs were used to examine main effects. We also performed multivariate analysis of variance (MANOVA) on multivariate fish and crayfish total growth (delta mass, delta length) and benthic community structure (Chironimid density, AFDM, and autotrophic index) variables. Shallow and deep habitats were examined separately for periphyton response variables, leaf litter decomposition, and macroinvertebrate density. All statistical assumptions were checked using residuals plots and appropriate diagnostic tests [67]. Statistical analyses were performed using SYSTAT version 13 (SYSTAT Software, San Jose, California) and R version 4.0.5 (R Core Development Team, 2021) with an α of 0.05.

## Results

### Drought

**Fig 1:**
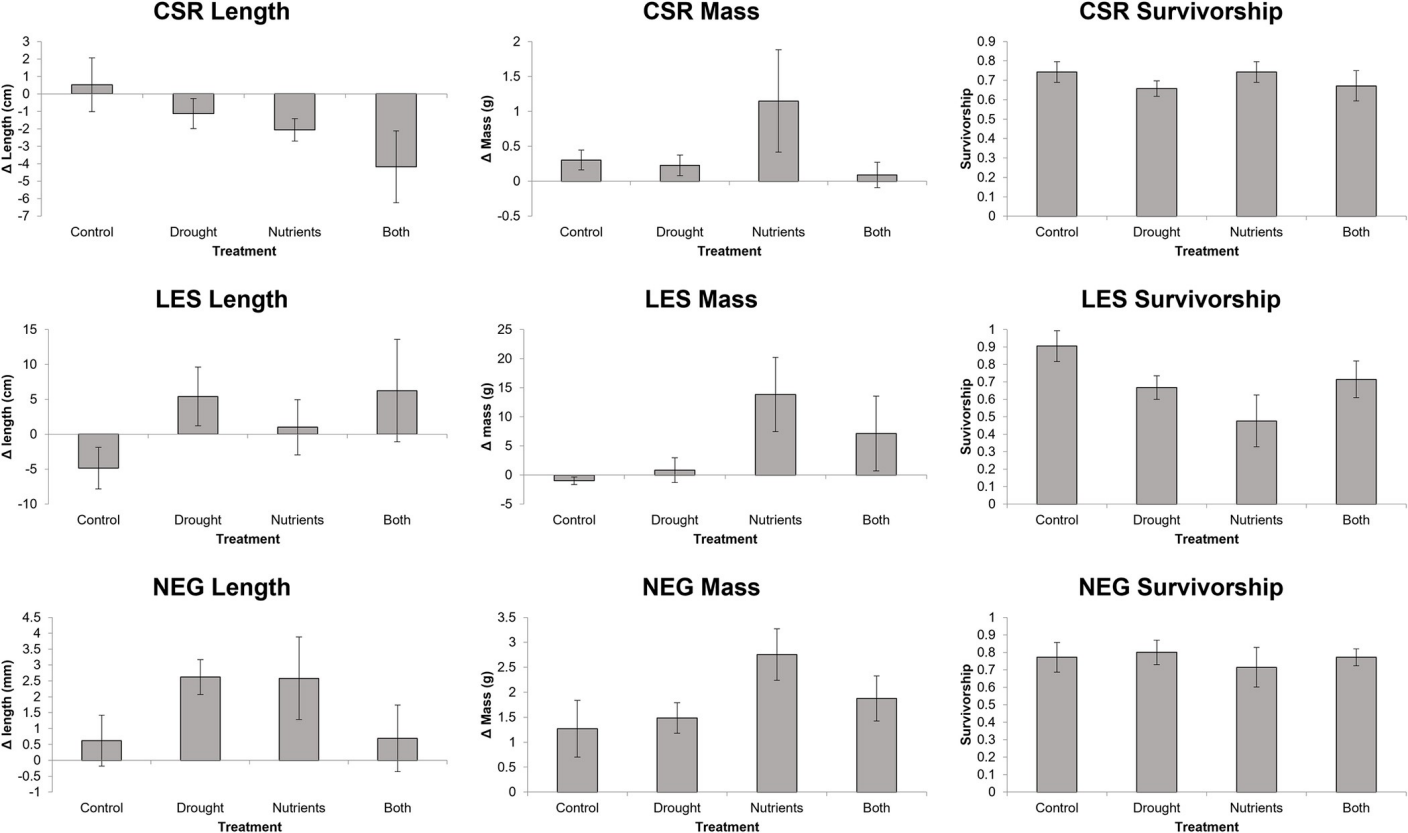
Change in length (left), mass (middle column), and survivorship (right) for Central Stonerollers (CSR, top), Longear Sunfish (LES, middle row) and Ringed Crayfish (NEG, bottom). Error bars are standard error.

**Fig 2:**
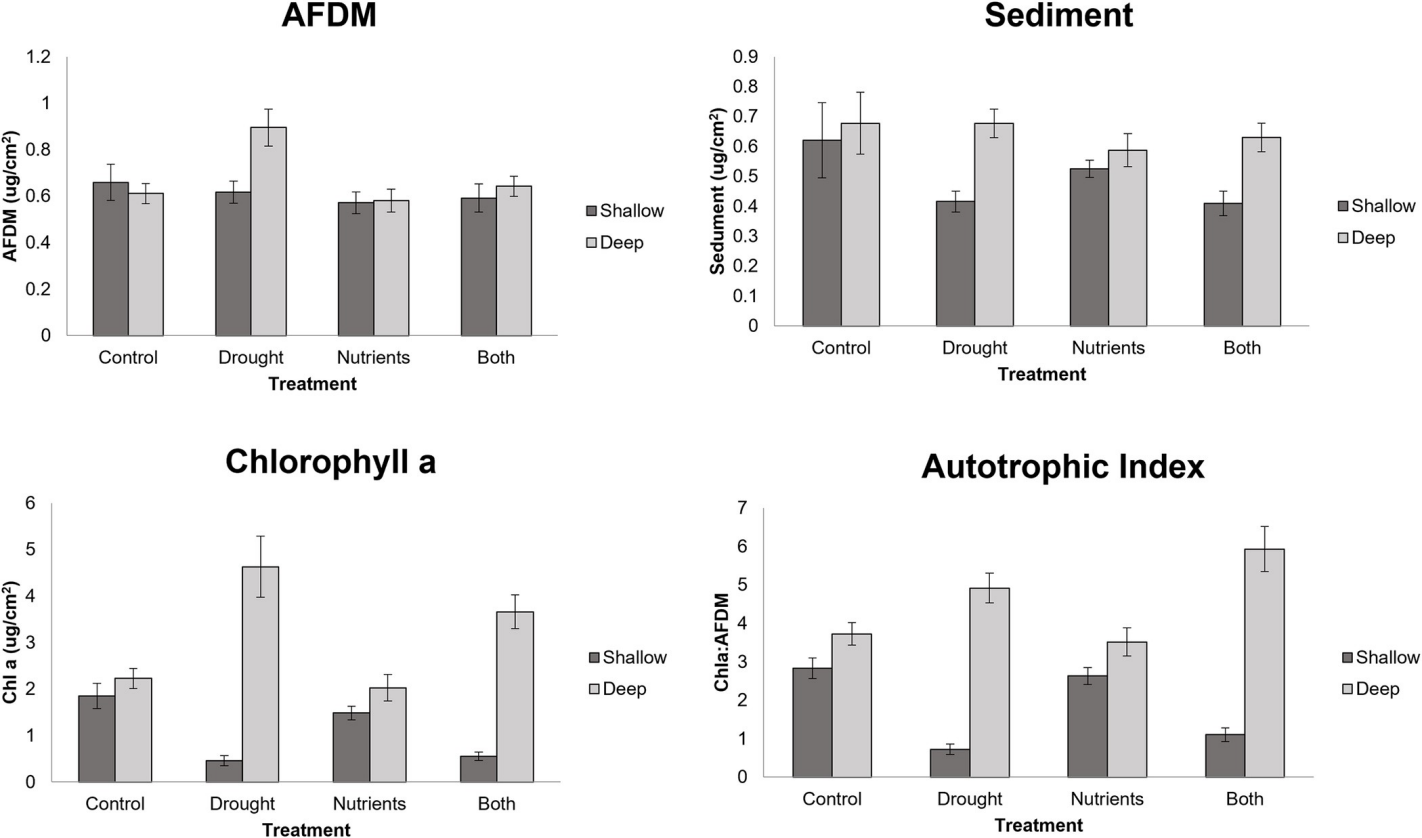
Ash free dry mass (AFDM, top left), Sediment levels (top right), Chlorophyll a (Chl a, bottom left), and Autotrophic Index (bottom right) for shallow and deep habitats. Error bars represent standard error.

**Fig 3:**
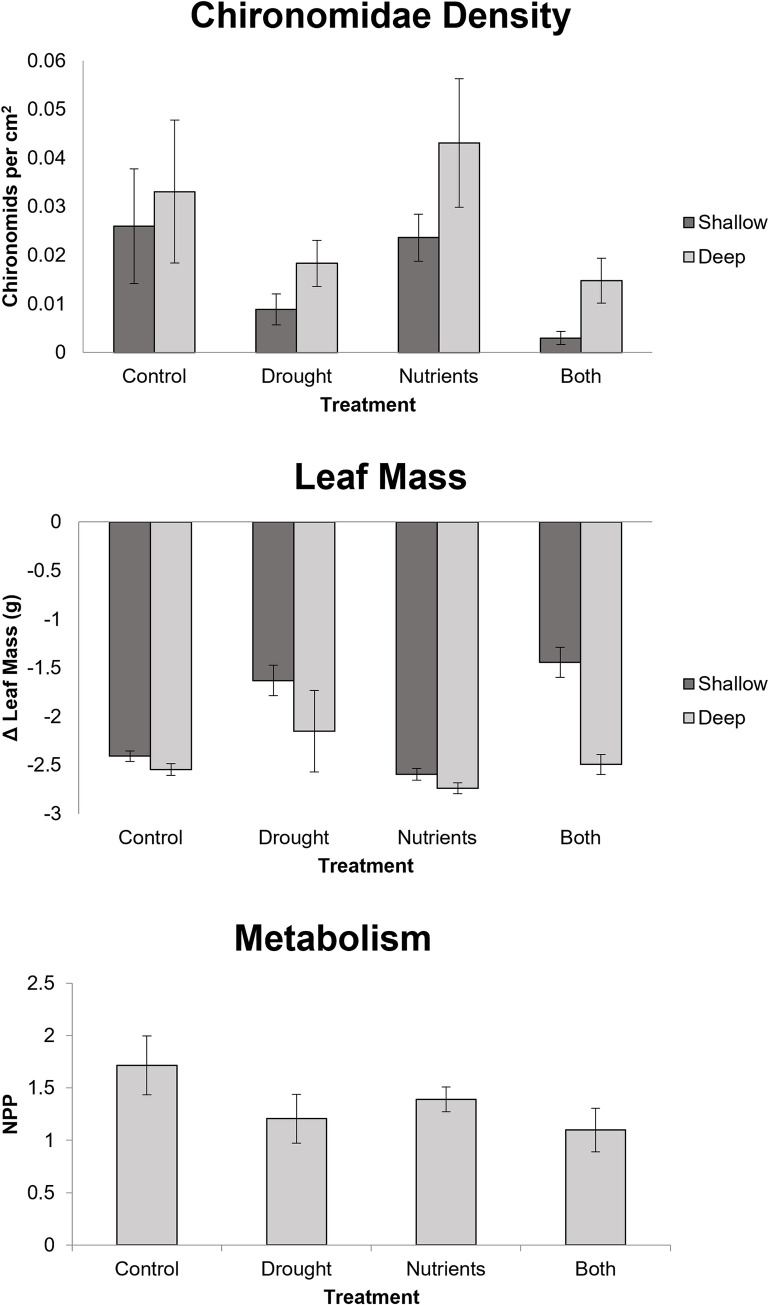
Chironomid density (top) and change in leaf mass (middle) for shallow and deep habitats; net primary production (bottom). Error bars represent standard error.

Drought affected fish and crayfish differentially. For central stonerollers, we found no significant main effect for drought for delta length, delta mass, or survivorship variables (Fig 1, Table 1). For longear sunfish, we saw a significant positive multivariate effect of drought on growth variables (Fig 1, Table 1). For ringed crayfish, we observed no effect of drought. (Fig 1, Table 1). In shallow habitats, drought had a negative effect on multivariate periphyton structure, as well as negative univariate effects on chlorophyll a concentration, sediment levels, chironomid density, and autotrophic index (Figs 2 and 3, Table 2). Drought had a negative influence on leaf litter decomposition in shallow habitats (Fig 3). In deep habitats, drought had an overall positive effect on multivariate benthic community structure (via increased autotrophic index and AFDM), as well as positive univariate effects on chlorophyll a concentrations, ash free dry mass, and the autotrophic index (Fig 2, Table 2). We did not observe any effect of drought treatment on net primary production or leaf decay in deep habitats. (Fig 3).

**Table 1:** Probability values associated with multivariate analysis of variance (MANOVA) for the effect of drought and nutrient enrichment on multivariate change of growth (change in mass and length) and analysis of variance (ANOVA) for the effect of drought and nutrient enrichment on change in length, change in mass, and survival of Central Stoneroller (CSR), Longear Sunfish (LES), and Ringed Crayfish (NEG).

Variable and Species	Multivariate effect (growth)	ΔMass	ΔLength	Survivorship
*CSR*				
Drought	F_2,23_ = 1.518, p = 0.240	F_1,24_ = 1.801, p = 0.192	F_1,24_ = 1.557, p = 0.224	F_1,24_ = 1.606, p = 0.217
Nutrients	F_2,23_ = 2.111, p = 0.144	F_1,24_ = 0.702, p = 0.410	F_1,24_ = 3.499, p = 0.074	F_1,24_ = 0.013, p = 0.909
Drought x Nutrients	F_2,23_ = 0.648, p = 0.532	F_1,24_ = 1.345, p = 0.257	F_1,24_ = 0.024, p = 0.879	F_1,24_ = 0.013, p = 0.909
*LES*				
Drought	**F**_**2,23**_ **= 3.897, p = 0.035**	F_1,24_ = 0.505, p = 0.484	F_1,24_ = 2.135, p = 0.157	F_1,24_ = 0.000, p = 0.999
Nutrients	F_2,23_ = 2.445, p = 0.109	F_1,24_ = 3.935, p = 0.059	F_1,24_ = 0.396, p = 0.535	F_1,24_ = 2.743, p = 0.111
Drought x Nutrients	F_2,23_ = 0.176, p = 0.839	F_1,24_ = 0.363, p = 0.552	F_1,24_ = 0.223, p = 0.641	**F**_**1,24**_ **= 4.286, p = 0.049**
*NEG*				
Drought	F_2,23_ = 0.558, p = 0.580	F_1,24_ = 0.425, p = 0.521	F_1,24_ = 0.003, p = 0.958	F_1,24_ = 0.635, p = 0.635
Nutrients	**F**_**2,23**_ **= 3.893, p = 0.035**	F_1,24_ = 3.412, p = 0.077	F_1,24_ = 0.000, p = 0.985	F_1,24_ = 0.231, p = 0.635
Drought x Nutrients	F_2,23_ = 1.806, p = 0.187	F_1,24_ = 1.158, p = 0.293	F_1,24_ = 3.472, p = 0.075	F_1,24_ = 0.874, p = 0.874

Bold highlights significant values (p< 0.05 for ANOVAs; Pillai’s Trace test used for MANOVAs).

**Table 2:** Probability values associated with multivariate analysis of variance (MANOVA) and analyses of variance (ANOVA) for effects of drought and nutrient enrichment on benthic community variables (chironomid density, ash-free dry mass [AFDM], autotrophic index [AI]).

Variable and Habitat	Community	Chl a	AFDM	Chironomids	AI	Sediment	Leaves
*Shallow Habitats*							
Drought	F_3, 50_ = 24.24, p **< 0.001**	**F**_**1, 52**_ **= 31.41, p < 0.001**	F_1, 52_ = 0.030, p = 0.863	**F**_**1, 52**_ **= 7.769, p = 0.007**	**F**_**1, 52**_ **= 72.01, p <0.001**	**F**_**1, 52**_ **= 4.909, p = 0.031**	**F**_**1, 52**_ **= 63.188, p <0.001**
Nutrients	F_3, 50_ = 0.406, p = 0.749	F_1, 52_ = 0.001, p = 0.974	F_1, 52_ = 0.830, p = 0.366	F_1, 52_ = 0.372, p = 0.545	F_1, 52_ = 0.173, p = 0.679	F_1, 52_ = 0.498, p = 0.483	F_1, 52_ = 0.000, p = 0.999
Drought x Nutrients	F_3, 50_ = 0.816, p = 0.491	F_1, 52_ = 2.585, p = 0.114	F_1, 52_ = 0.258, p = 0.615	F_1, 52_ = 0.068, p = 0.795	F_1, 52_ = 1.862, p = 0.178	F_1, 52_ = 0.377, p = 0.542	F_1, 52_ = 2.344, p = 0.132
*Deep Habitats*							
Drought	**F**_**3, 50**_ **= 8.199, p < .0001**	**F**_**1, 52**_ **= 22.03, p <0.001**	**F**_**1, 52**_ **= 8.959, p = 0.004**	F_1, 52_ = 3.968, p = 0.052	**F**_**1, 52**_ **= 16.923, p<0.001**	F_1, 52_ = 0.089, p = 0.766	F_1, 52_ = 1.960, p = 0.167
Nutrients	**F**_**3, 50**_ **= 3.593, p = 0.019**	F_1, 52_ = 1.849, p = 0.180	**F**_**1, 52**_ **= 6.027, p = 0.017**	F_1, 52_ = 0.090, p = 0.765	F_1, 52_ = 0.840, p = 0.364	F_1, 52_ = 0.959, p = 0.332	F_1, 52_ = 1.392, p = 0.243
Drought x Nutrients	**F**_**3, 50**_ **= 2.902, p = 0.044**	F_1, 52_ = 0.790, p = 0.378	F_1, 52_ = 3.709, p = 0.060	F_1, 52_ = 0.394, p = 0.533	F_1, 52_ = 1.948, p = 0.169	F_1, 52_ = 0.097, p = 0.757	F_1, 52_ = 0.079, p = 0.742

Bold highlights significant values (p< 0.05 for ANOVAs; Pillai’s Trace test used MANOVAs).

### Nutrients

For ringed crayfish, we observed a significant positive effect of nutrients on multivariate growth, but no additional main effects (Fig 1, Table 1). Additionally, central stonerollers and longear sunfish had no observable nutrient main effects (Fig 1, Table 1). We observed no nutrient main effects on benthic variables in shallow habitats (Figs 2 and 3, Table 2). However, nutrients had a negative effect on ash free dry mass in deep habitats, but an overall positive influence on multivariate periphyton variables by increasing the autotrophic index (Fig 2, Table 2). Nutrients did not affect ecosystem functioning metrics (Fig 3).

### Interactive effects

For longear sunfish, we saw a significant a univariate, antagonistic interaction of growth and nutrient treatments on survivorship (Fig 1, Table 1). Additionally, drought and nutrient pollution interacted synergistically to influence multivariate benthic community structure in deep habitats (Figs 2 and 3, Table 1). We saw no additional interactive effects on any variable.

## Discussion

We found that drought, nutrients, and the combined effects of these stressors affected many aspects of stream ecosystem structure and functioning. While the inherent environmental heterogeneity of lotic systems might foster a greater potential for evolutionary adaptations to multiple stressors [9,16], our study found that stressor interactions were highly context dependent, and differentially impacted ecosystem structure and function based largely on trophic position. Though many examinations of multiple stressor systems focus on population-level responses [14], community and ecosystem-scale examinations can elucidate the impact of individual and multiple stressors, even if individual population responses are obscured [68,69].

### Drought

Our study found that drought negatively influenced many portions of the benthic community in shallow habitats. As in real systems exposed to drying, our tiles and leaf packs were above the waterline for portions of the experiment, and these results coincide with previous examinations of drought systems. Previous field [32,33,70] and mesocosm [52,57] studies have shown that drought can negatively affect algal communities [71,72]. Additionally, drought can negatively impact macroinvertebrate taxa [30,73], cause a decrease in the autotrophic index as photosynthetic components of the periphyton die [52], and slow the decomposition of leaf litter [52,71]. While previous research has connected drought to increased levels of sedimentation (e.g., [74]), drought decreased sediment levels in our study. However, closed-system mesocosm experiments that examine drought might not allow sufficient time for sediment accumulation prior to water withdrawals [57].

Surprisingly, drought treatments positively affected chlorophyll a concentrations, ash free dry mass, and the autotrophic index in deep habitats. Many algal species often persist in pool refuges during drought, and pool habitats can provide important source populations for algal recolonization after drought events [75]. As our drought treatments included wetted pools, they might have served as refuges for algal species in our experiment and facilitated their population growth. Drought decreased—though not significantly—chironomid densities (p = .052) in deep habitats. Concentration effects may have increased the predatory influences of longear sunfish and ringed crayfish on chironomids, causing a top-down cascade that released algal communities from grazing pressures [52,76]. Previous work has demonstrated that grazing pressures can influence algal community structure [77] and grazing during droughts can further alter periphyton composition [32,78]. Increased grazing by fish species might also reduce the amount of senescent algal cells, increasing chlorophyll a concentrations [76,79,80]. Potential alterations of periphyton community structure coupled with a suppression of macroinvertebrate grazers might explain the positive effect of drought on periphyton in our study. Additionally, reduced water levels might have concentrated available nutrients, further facilitating algal growth.

Drought also impacted some aspects of ecosystem functioning in our experiment. Previous work has shown that drought can negatively impact leaf breakdown [52,71], and our drought treatments produced similar patterns. Net primary production was unaffected by drought treatment. Similar studies have shown drought negatively impacts NPP [52,72]. However, our experimental design necessitated that the pump output provide minor (but unmeasured) supplemental aeration to prevent complete fish mortality. Though all experimental tanks were equally aerated, the increase in dissolved oxygen might have reduced our ability to observe changes in NPP when compared to natural systems.

Fish and crayfish were largely drought tolerant in our study. Our focal species are native to the Ozark region of northwestern Arkansas [60]. Streams in this region are prone to seasonal drying and desiccation [7] and it is likely that many fish and crayfish in this region have evolved adaptations to drought events [25]. Surprisingly, longear sunfish growth was positively affected by drought conditions in our study. Because drought positively affected many aspects of the benthic community, we suspect longear sunfish growth was supplemented by increased resource availability. This result contrasts previous work that showed that drought can have a negative impact on fish and crayfish body size [57,81,82]. Furthermore, previous studies [e.g. 83,84] have demonstrated that density-dependent factors can negatively affect growth and survivorship of fish and crayfish. Because drought treatments increased relative density, we anticipated density-dependent responses [57]. However, bottom-up increases in resources—and increased feeding on macroinvertebrate prey items—in drought tanks might have been sufficient to ameliorate any density-dependent effects. However, as we only recorded mean length and mass at the beginning and end of the experiment, some treatment effects might be masked by size-based mortality—though we did not observe any apparent size bias. Additionally, any aeration of the tanks provided by our filtration pumps might artificially inflate survivorship when compared to natural settings.

### Nutrients

Nutrient treatments caused a net increase in mass for all species, however, only ringed crayfish showed statistically significant differences in growth resulting from nutrient additions. Bottom-up trophic cascades are well documented in the literature (e.g., [85]), and nutrient additions can cause an overall increase in resource availability if the system is nutrient-limited [40,86]. In our study, nutrient addition altered multivariate benthic community structure in deep habitats. Though we did not see a univariate influence of nutrients on chironomids, we did observe a multivariate change in the benthic community—including an increase chironomid density. Additionally, the positive effect of nutrients on crayfish growth and the subsequent increase in grazing on both algae and chironomids that led to a decrease of ash free dry mass suggests cascading trophic interactions. Previous work has demonstrated that an increase in biomass at higher trophic levels can affect the algal resources even in nutrient enriched systems [87]. As crayfish both directly consume algal stocks, and feed on chironomid grazers [87], they can impact food web structure across multiple trophic levels—which could explain their nutrient response. Central stonerollers showed no nutrient response in our treatments. While both central stonerollers [88] and ringed crayfish [52] can influence algal communities, crayfish are a key driver of ecosystem processes in stream systems [89] and can process larger quantities of algal and detrital biomass than stonerollers [90].

### Combined effects

Drought and nutrient treatments interacted antagonistically on longear sunfish survival. Nutrients caused a non-significant net increase in longear sunfish biomass (p = .059), which likely increased competition for food resources as energetic requirements increased, thus negatively influencing survival rates. However, the increased resource availability of drought treatments ameliorated competitive interactions and increased survival. Drought and nutrient treatments interacted synergistically in shaping multivariate periphyton structure—causing a multivariate increase in photosynthetic portions of the periphyton (autotrophic index). The suppression of chironomids by increased predation pressures during drought (indicated by shifts in multivariate community structure), the potential concentration of nutrients in drought treatments, and the net increase in photosynthetic portions of the periphyton resulting from nutrient additions synergistically altered basal resources. While these effects positively influenced fish and crayfish species in our study, previous work has shown that drought and nutrient pollution might interact to negatively influence population dynamics [24]. However, negative effects of nutrient enrichment tend to be more influential over longer time-scales than our experiment examined [17,48,91]. Our results reinforce the findings of other studies (e.g., [48,92]) that demonstrate drought and nutrient pollution can influence ecosystem structure and function in aquatic systems. While the drought-mediated increases in periphyton variables and increased grazing on chironomids helped to ameliorate some negative effects of nutrient enrichment on fish and crayfish populations, the relationship between drought and nutrient pollution on periphyton variables might have long term impacts on population and community dynamics. Furthermore, increased drought severity, increased nutrient levels, or longer observational scales would likely elicit different effects than the ones our experiment produced. Accordingly, additional examination of how these stressors interact in a variety of systems is warranted.

Our work highlights the complexity of drought and nutrient effects on stream communities and emphasizes that our understanding of individual stressors might not adequately inform our knowledge of multi-stressor systems. Because anthropogenic development and resource use is likely to increase the relative effects of drought and nutrient pollution on stream systems, additional experimental, observational, and modelling work is necessary to clarify the mechanisms of compounded stressors. As individual stressors can differentially interact on varying aspects of the food web simultaneously, management or conservation strategies that incorporate only one stressor might be inadequate to conserve stream systems.
